# An evaluation of the effects of localised skin cooling on microvascular, inflammatory, structural, and perceptual responses to sustained mechanical loading of the sacrum: A study protocol

**DOI:** 10.1371/journal.pone.0303342

**Published:** 2024-05-10

**Authors:** Ralph J. F. H. Gordon, Peter R. Worsley, Davide Filingeri

**Affiliations:** 1 ThermosenseLab, Skin Sensing Research Group, School of Health Science, University of Southampton, Southampton, United Kingdom; 2 PressureLab, Skin Sensing Research Group, School of Health Science, University of Southampton, Southampton, United Kingdom; PLOS: Public Library of Science, UNITED KINGDOM

## Abstract

This study protocol aims to investigate how localised cooling influences the skin’s microvascular, inflammatory, structural, and perceptual tolerance to sustained mechanical loading at the sacrum, evaluating factors such as morphology, physiology, and perceptual responses. The protocol will be tested on individuals of different age, sex, skin tone and clinical status, using a repeated-measure design with three participants cohorts: i) young healthy (n = 35); ii) older healthy (n = 35); iii) spinal cord injured (SCI, n = 35). Participants will complete three testing sessions during which their sacrum will be mechanically loaded (60 mmHg; 45 min) and unloaded (20 min) with a custom-built thermal probe, causing pressure-induced ischemia and post-occlusive reactive hyperaemia. Testing sessions will differ by the probe’s temperature, which will be set to either 38°C (no cooling), 24°C (mild cooling), or 16°C (strong cooling). We will measure skin blood flow (via Laser Doppler Flowmetry; 40 Hz); pro- and anti-inflammatory biomarkers in skin sebum (Sebutape); structural skin properties (Optical Coherence Tomography); and ratings of thermal sensation, comfort, and acceptance (Likert Scales); throughout the loading and unloading phases. Changes in post-occlusive reactive hyperaemia will be considered as the primary outcome and data will be analysed for the independent and interactive effects of stimuli’s temperature and of participant group on within- and between-subject mean differences (and 95% Confidence Intervals) in peak hyperaemia, by means of a 2-way mixed model ANOVA (or Friedman). Regression models will also be developed to assess the relationship between absolute cooling temperatures and peak hyperaemia. Secondary outcomes will be within- and between-subject mean changes in biomarkers’ expression, skin structural and perceptual responses. This analysis will help identifying physiological and perceptual thresholds for the protective effects of cooling from mechanically induced damage underlying the development of pressure ulcers in individuals varying in age and clinical status.

## Introduction

Pressure ulcers (PUs) are localised damage to the skin and sub-dermal tissues, resulting from sustained periods of pressure, or pressure in combination with shear forces [1]. In the United Kingdom alone, the annual cost of treating chronic wounds, including PUs, has been estimated at £8.3 billion [2]. Accordingly, an improved understanding of the fundamental mechanisms underlying the physiological tolerance of human skin to mechanical loading could lead to the development of cost-effective, personalised solutions to prevent these wounds and improve patient care and quality of life.

Sustained localised mechanical loading on the skin can arise from lying and sitting postures, as well as the prolonged attachment of medical devices, e.g. prosthetics or respiratory masks [3, 4]. Internal tissue deformations will occur as a result of sustained pressure and shear forces that can lead to changes in the physiology of skin and sub-dermal tissue, including ischemia in the blood vasculature, lymphatic impairment, and direct deformation damage [5]. When load is removed, ischemia reperfusion injury may also occur due to the onset of post-occlusive reactive hyperaemia [6]. Reactive hyperaemia can increase the risk of ischemia reperfusion injury by triggering the release of oxygen-derived free radicals with cytotoxic effects, and this can play a role in the pathophysiology of PUs [7]. In addition, microclimate conditions within and around skin tissues strongly influence its tolerance to mechanical loading. For example, elevated temperature and humidity at the skin interface reduces the mechanical stiffness and strength of the skin and can increase its friction coefficient [4]. In contrast, cooling reduces skin tissue’s metabolic demands and could increase the skin’s physiological tolerance to mechanical damage [4, 8].

Evidence that changes in skin temperature could play a role in the tolerance of the skin to mechanical loading and shear came from early animal studies using porcine models, revealing that reduced skin temperature minimises the risk of PU formation through altered microvascular responses [9, 10]. More recently, the protective effective of reducing skin temperature has been demonstrated in rats [11, 12]. While this evidence highlights the potential therapeutic role of skin cooling for protecting tissue health, the mechanisms by which cooling enhances skin tolerance to pressure remain poorly understood in humans [9–11, 13–16]. Specifically, it remains unclear whether and to what extent the benefits of lowering skin temperature arise from the individual or combined effects of: 1) preserved microvascular function during mechanical loading and/or attenuated post-occlusive reactive hyperaemia following on pressure release; and 2) downregulation of skin’s inflammatory responses to sustained mechanical pressure. Animal studies revealed that local cooling, as well as the stimulation of cold sensitive TRPM8-expressing neurons in dorsal root ganglions, could modulate the skin’ inflammatory responses to acute mechanical stress (e.g. pressure loading) [12] and chronic skin damage (e.g. chronic dermatitis) [17], via downregulation of the expression of pro-inflammatory cytokines such Tumour Necrosis Factor alpha (TNF-α).

In addition to its physiological effects, it is well known that localised cooling of the skin can induce cold discomfort, which, if the magnitude of the cooling stimulus is large enough, can limit acceptability and adherence to therapeutic interventions designed to maintain skin health, particularly for vulnerable individuals at risk of PUs such as the elderly [18]. Hence, cold-induced discomfort could provide an obstacle for the adoption of skin cooling as a therapeutic intervention to promote skin integrity in humans. However, there is limited evidence on how the absolute cooling temperature and applied pressure on the skin interact in driving discomfort [19]. Despite these challenges, several support surfaces and therapeutic interfaces have been designed with local and full body cooling applied through microclimate management systems [20–22], although the evidence underlying their efficacy remain limited.

Modelling the relationship between the physiological and perceptual effects of skin cooling could provide an empirical approach to identify a common level of cooling that proves both physiologically beneficial and perceptually acceptable. This could then be translated to inform design parameters for more effective support surfaces and therapeutic interfaces.

It should also be recognised that the underlying tolerance to pressure at the skin interface, as well as the physiological and perceptual effects of cooling to pressure-induced damage, may vary as a function of age and comorbidities [3]. These states are associated with changes in skin biophysics and morphology [23], and in thermoregulatory and perceptual sensitivities. For example, ageing is likely to modulate the effects of cooling on tissue tolerance, as aged skin presents a reduced physiological and perceptual sensitivity to cold, due to decreases in both reflex cutaneous vasoconstriction, and density of thermoreceptors [24]. Similarly, the presence of a spinal cord injury (SCI) is associated with autonomic (e.g. impaired control of skin blood flow) and sensory dysfunctions (e.g. perceptual loss below injury level) [25, 26]. Thus, there may be variations between reductions in cold sensitivity and diminished efficacy of therapeutic cooling associated with age and comorbidities.

This study protocol aims to investigate: 1) how different levels of localised cooling influences the skin’s microvascular, inflammatory, structural, and perceptual responses to sustained mechanical loading at the sacrum; and 2) how aging and spinal cord injury may modulate the metabolic, immunological, biophysical, and perceptual pathways underlying the beneficial effects of localised cooling on skin tolerance to mechanical loading. To achieve our aims, we have designed a clinically relevant experiment in healthy young participants and in groups at-risk of PUs (i.e., older and SCI), as detailed in the sections below. The investigation will offer a combination of skin viability, thermal physiology, and non-invasive skin sensing technologies, to develop new basic knowledge on the role of temperature in reducing the risk of skin damage. This will support innovation in the design of healthcare and user-centred technologies, such as mattresses, clothing, and medical devices that can safely interface with the skin and provide some protection from damage. This will unlock the potential of cooling to the skin that will help maintain skin health across the life course.

## Materials and methods

### Overview

**Fig 1:**
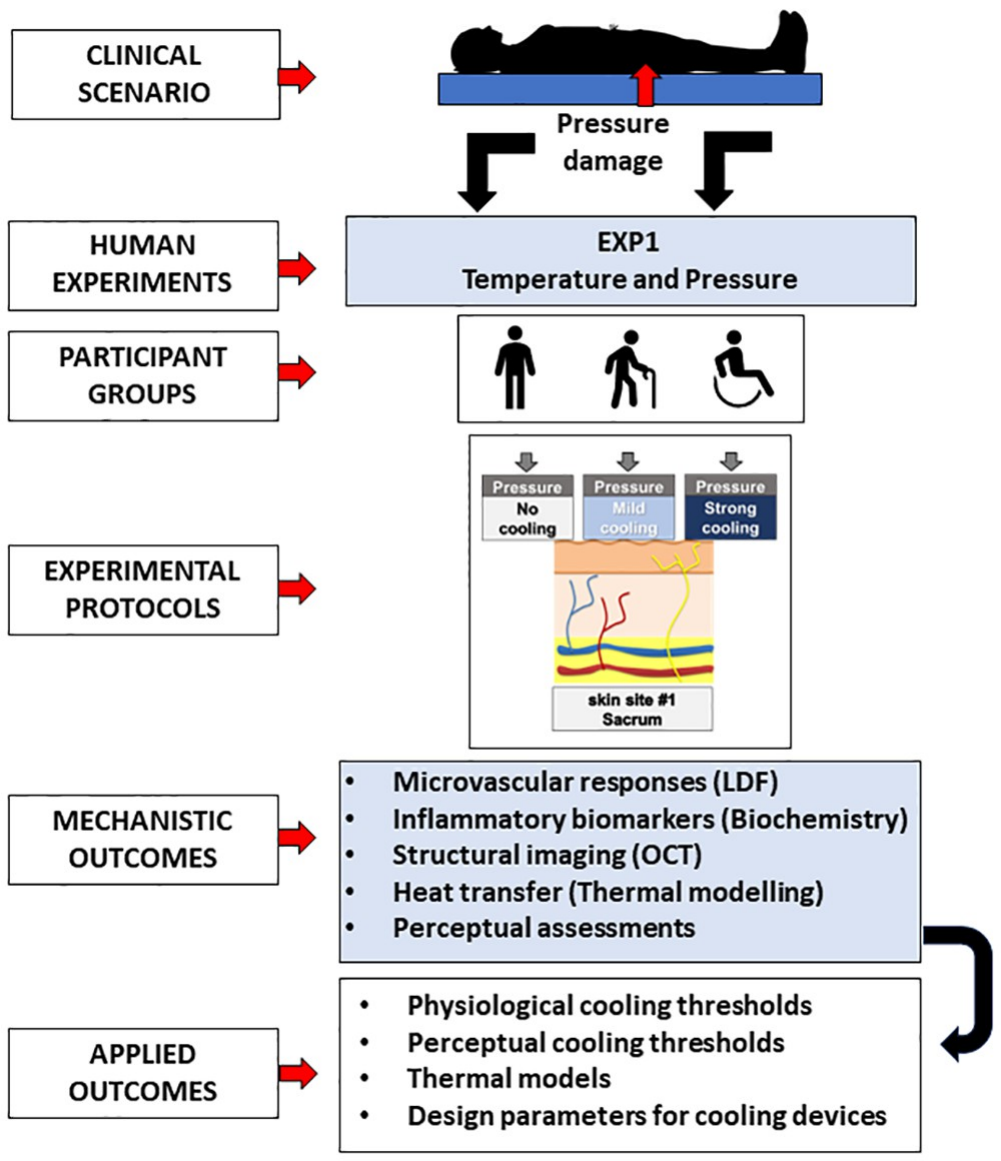
Experimental design. We have designed a clinically relevant experiment in healthy young participants and in groups at-risk of PUs (i.e., elderly and spinal cord injured, SCI), which will determine how different levels of cooling [i.e. no cooling (38°C), mild cooling (16°C), and strong cooling (16°C)] alter the skin’ microvascular, inflammatory, structural, and perceptual responses to sustained pressure-induced ischemia and reactive hyperaemia. From an applied standpoint, the research will identify physiological and perceptual cooling thresholds (i.e. level of cooling, modulations via age and clinical status), which could be used as design parameters for the development of user-centred medical devices and thermal wearables. LDF: Laser Doppler Flowmetry; OCT: Optical Coherence Tomography.

Participants will attend the laboratory within the Clinical Academic Facility located at Southampton General Hospital (Southampton, UK), to complete three experimental sessions separated by a minimum of 24 hrs. During the sessions, participants’ skin over the sacrum will be mechanically loaded (60 mmHg; 45 min) and unloaded (minimum tare load: 17.5 mmHg; 20 min) with a custom-built thermal probe, to cause pressure-induced ischemia and post-occlusive hyperaemia. The study will be a randomised cross-over design, involving three probe temperatures, which will be set to either 38°C (no cooling), 24°C (mild cooling), or 16°C (strong cooling). An overview of the study design can be found in Fig 1.

### Participants

Three participant cohorts will be recruited: i) young healthy (n = 35); ii) older healthy (n = 35); and iii) spinal cord injured (SCI, n = 35). Sample size calculations were performed using Gpower (Gpower 3.1) with an effect size f = 0.4 for a repeated-measure ANOVA [parameters: within-between interaction; α = 0.05; β = 0.80; 3 groups; 3 measurements (i.e. peak hyperaemia at 38, 24, 16°C)], based on published data on the mean difference in peak hyperaemia (expressed as percentage of maximal cutaneous vascular conductance, CVCmax) during control conditions (32.7 ± 9.4% CVCmax) and during sensory nerve block (17.3 ± 6.8% CVCmax) [27]. Changes in peak hyperaemia were identified as the primary experimental outcome, as this represents a robust and repeatable microvascular response. The effects of cooling on peak hyperaemia are likely to be similar to those of cutaneous sensory nerve bloc [27], given that cooling also impairs the activity of cutaneous sensory nerves [28]. Hence, it was identified that the 50%-reduction in peak hyperaemia reported by Lorenzo and Minson [27] was a large (f = 0.4) and physiologically meaningful effect size to evaluate the beneficial effects of cooling on sustained mechanical loading. Based on the data above, we estimated a minimum sample size of 18 participants per group, and we propose testing of 35 individuals per group to allow for sufficient statistical power and to account for up to 50% dropout. Participants will be recruited according to the criteria in Table 1.

**Table 1:** Participant inclusion and exclusion criteria.

Inclusion	Exclusion
18–70 years old (young healthy 18–35 years old; older healthy 55–70 years old).	Young and older health groups only (does not apply to SCI group, see text below): suffering from cardiovascular, metabolic, and neurological disorders and/or comorbidities, e.g., hypertension, diabetes, chronic lung disease.
Male or female (minimum 17 for each sex).	Raynaud’s disease.
Skin tone dark, medium, or light (minimum 11 for each tone—assessed via the Fitzpatrick Scale [29]).	Suffering from skin conditions (e.g., eczema).
Healthy groups only: physically active (i.e., performing exercise 1 to 3 times a week).	Under drug therapy affecting thermoregulation (e.g. muscarinic antagonists).
SCI group only: presenting thoracic injury/paraplegia (i.e., injury level within T1-S1).	Smoker or Vaper.
SCI group only: >12 months post-injury and no history of PUs.	

As identified by the “Guidelines for the conduct of clinical trials for spinal cord injury as developed by the ICCP Panel” [30], inclusion and exclusion criteria for SCI participants should consider the confounding effects of various independent variables such as pre-existing or concomitant medical conditions, other medications, surgical interventions, and rehabilitation regimens. As it may not be practical or justifiable to limit study enrolment based on factors such as e.g., rehabilitation regime or sex, all potentially confounding factors will be comprehensively recorded and considered as potential co-variables in primary and secondary data analyses.

### Experimental procedures

Once screened and recruited, participants will be invited to their pseudo-randomly allocated experimental session [31]. They will come to the laboratory wearing comfortable, loose-fitting attire. Upon arrival, participants will be seated whilst they acclimatise to the ambient conditions of the laboratory (22–24 °C; 50% RH) before recording height and body mass (Model 874; Seca GmbH, Hamburg, Germany).

Following the pre-experimental checks, participants will lie down in the prone position on a hospital bed. Care will be taken to ensure that participants are as comfortable as possible by providing pillows as necessary to support the pelvic region and upper body, given the length of time (75 minutes) they will need to remain in this position. The addition of the pillows also serves to support the lumbar region by flattening the sacroiliac joint and reducing any pronounced lordosis of the spine while lying in a prone position.

First, structural, and functional imaging of the skin of the sacral skin using Optical Coherence Tomography (OCT) will be performed. This will be followed by sampling of skin sebum at the sacrum for subsequent biomarker analysis, using an established methodology involving a 2-minute application time and tweezer extraction to avoid cross-contamination [32]. The investigator will then place a custom-built thermal probe over the sacrum and load it with a weight (see below for details) to achieve pressure of 60 mmHg. The thermal probe consists of a water-perfused set of Peltier elements, which provide local temperature control for a 36-cm^2^ plate. An optic fibre is integrated in the plate, flush to its surface, which allows for the continuous monitoring of skin blood flow via Laser Doppler Flowmetry (LDF). The thermal probe is mounted on a frame with an integrated strain gauge to estimate the force applied to the stimulator. When applied to the skin, the integrated device allows for the manipulation of local skin temperature (range: 0°C to >50°C; variable temperature rates under PID control), applied pressure and the concurrent monitoring of blood flow.

To ensure consistent placement between sessions, probe placement will be marked with non-permanent ink. To minimize edge loading, the thermal probe is equipped with a 3D printed sleeve (thermoplastic polymer (Poly lactic acid)) and to ensure uniform pressure distribution across the mechanically loaded sacrum an interface pressure mapping device will be used (ForeSiteSS, XSensor, Canada). The pressure mapping device comprises a 2*32 sensing array with a spatial resolution of 12.7mm, operating between 5-256mmHg, with an accuracy of ± 5%.

**Fig 2:**
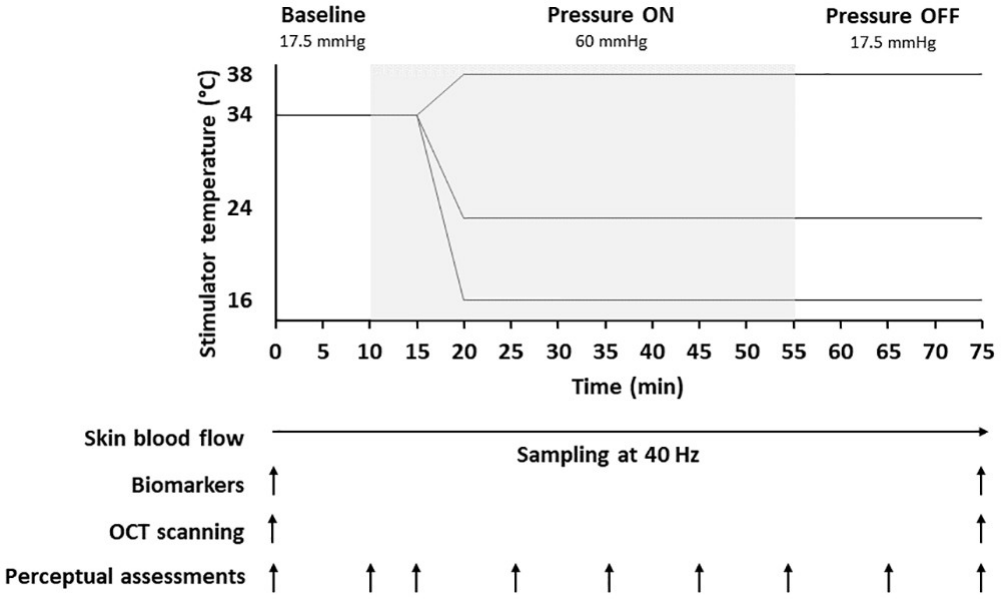
Standardise pressure protocol delivered over the sacrum. The thermal probe will be used to deliver a standardised 60mmHg pressure protocol to evoke pronounced tissue ischaemia under 3 thermal conditions, i.e. a control skin temperature evoking no cooling (i.e. 38°C) and two cooling temperatures of 24°C and 16°C. During the protocol, a series of non-invasive measurements will be conducted [i.e. skin blood flow via LDF; inflammatory biomarker sampling from skin sebum; structural and functional imaging via Optical Coherence Tomography (OCT); perceptual assessment of subjective thermal sensation, comfort, and acceptance] at different time points (identified in the diagram by ↑).

Finally, participants will be asked to provide subjective ratings of thermal sensation, comfort, and acceptability, using Likert scales (detailed below). At this point, the standardised protocol to cause pressure-induced ischemia and post-occlusive hyperaemia will commence (Fig 2).

The protocol consists of three phases; i) a 10-minute baseline stabilisation with minimal pressure (17.5 mmHg (2.3 kPa)), ii) 45-minute loading phase (60 mmHg/7.9 kPa [15)), and iii) a 20-minute minimal pressure phase (same as baseline). Skin blood flow will be measured continuously throughout the three phases of the protocol via Laser Doppler Flowmetry (Moor Instruments, moorVMS-LDF laser Doppler monitor, UK) with perceptual responses measured at predetermined intervals during the protocol (Fig 2). During the baseline phase in all conditions, the temperature probe will be set to 34 °C. On starting the loading phase, the thermal probe will be loaded with a 2 kg weight to elicit an equivalent pressure of 60mmHg (7.9 kPa) at the probe interface. The initial 5-mintues of the loading phase will serve as a further baseline measurement for the pressure protocol, to delineate the effects of 60mmHg loading alone on microvascular responses. To this end, once the load is applied to the sacrum, the probe temperature will remain at 34 °C. Following this, the temperature probe will be set to the target temperature for the specific testing session, either 38 °C (Δ13.7 °C/min), 24 °C (Δ11.3 °C/min), or 16 °C (Δ9.2 °C/min) and will be maintained at such temperature until the end of the session.

Upon completion of the loading phase, the thermal probe will be unloaded (Fig 2) whilst maintained in position over the sacrum, to allow skin tissue blood flow reperfusion (which will be continuously monitored by via LDF). The minimal loading phase (comparable to the initial 10-minute baseline phase) will last for 20 minutes, after which the thermal probe will be removed to allow for image acquisition via OCT and a final sampling of skin sebum over the sacrum.

The sections below provide a detailed description of each measurement undertaken during the protocol.

## Measurements

### Skin blood flow

Continuous skin blood flow will be monitored via Laser Doppler Flowmetry at the sacrum.

LDF is a non-invasive technique that uses optical probes to measure blood flow velocity in the microvasculature. Typically, tissue thickness is sampled at 1mm implementing the Doppler principle whereby light from a monochromatic laser becomes scattered from moving red blood cells, allowing it to be applied to a wide range of anatomical locations [33]. Given its relative low cost and ease of use, LDF is validated [34] and has been widely used to assess changes in blood flow velocity (as an index of changes in flow) over bony prominences, such as the sacrum [6, 13–15, 35].

The optical probe is integrated within the custom-built temperature probe, allowing concurrent manipulation of skin temperature whilst monitoring real-time changes in skin blood flow, during loaded and minimal loaded states. Blood flow during the loading phase will be analysed via spectral analysis of wavelet frequency to investigate temperature-modulated regulatory mechanisms during loading (i.e. changes in 0.1 Hz, 0.04 Hz, and 0.01 Hz frequencies will be associated with myogenic activity of vascular smooth muscles, neurogenic activity of the vessel wall, and vascular endothelium related metabolic activity, respectively) [11]. Blood flow during the minimal loading phase will be used to calculate the baseline skin blood flow (taken as the mean average during the final 3-minutes of the 10-mintue baseline period). The average baseline will then be used for normalisation of peak hyperaemia [(peak hyperaemia—average baseline skin blood flow)/average baseline skin blood flow)*100)] to investigate its modulation via cooling. Changes in peak hyperaemia represent a robust and repeatable microvascular response [36], which is directly implicated in the pathophysiology of PUs [36]. Secondary perfusion parameters will include the time to peak hyperaemic response and the perfusion area between the skin blood flow response curve and the mean baseline skin blood flow after unloading and during the reperfusion phase. In the measurement of peak hyperaemia skin blood flow will be collected continuously (40 Hz sampling rate) and averaged every 1-minute for the analysis of the temporal dynamics of cooling induced changes in skin blood flow under loading.

### Biomarkers

A preselected panel of pro- (IL-1α, IL-1β, TNFα, IL-6, IL-8, IFNγ) and anti-inflammatory biomarkers (IL-1RA) will be extracted from skin sebum. Biomarker collection and extraction techniques via application of Sebutape have been optimised in our laboratory [37], to ensure both low abundance and high abundance proteins can be quantified. In brief, Sebutape will be applied to the sacrum for 2-minutes before the samples are extracted using tweezers and a gloved hand to avoid cross contamination. Stored samples will be coded and stored at– 80 °C prior to analysis using standard ELISA plates for targeted proteins. The extraction of skin inflammatory biomarkers will use chemical and mechanical stimuli to for maximal extraction efficiency. Chemical extraction will involve 0.85 mL of extraction buffer, which consists of PBS + 0.1% Dodecyl maltoside. The tapes will then be shaken with the buffer for 1 hour followed by 5 minutes of sonication. A 0.35 mL aliquot will then be used for total protein analysis. The remaining 0.5 mL will be centrifuged for 10 minutes at a speed of 15 000g at 4°C. The supernatants will be discarded and the remaining solution with the pellet briefly vortexed and used for the immunoassay analysis, as prescribed by the manufacturer using MSD U-Plex kits (MesoScale Diagnostics).

### Skin imaging

Skin imaging via Optical Coherence Tomography using VivoSight^®^ device with dynamic OCT processing software (Michelson Diagnostics Ltd., Maidstone, Kent, UK) with a Class 1M (EN 60825–1) laser source of near-infrared wavelength (1305 nm). A total of 120 images with 50 μm spacing will be acquired as a 6 × 6 × 2 mm^3^ (width × length × depth) stack. This technique is non-inferior to punch biopsy for skin characterization [38] and will allow to non-invasively characterise the skin’s epidermal (i.e. thickness, stratum corneum hydration, collagen density) and blood perfusion properties (i.e. vascular plexus density and diameter) prior to and following the thermomechanical manipulations. This evaluation will also be relevant to model group differences in baseline skin anatomy and biophysics that may underlie the differential effects of cooling with ageing and SCI. The OCT probe will be placed gently on the skin, maintaining a static position during acquisition. Spacers at the probe interface will be used to optimise the focal point of the epidermis during scanning.

### Perceptual assessments

Perceptual assessments of participants’ local thermal and comfort sensations will be assessed via Likert scales, to establish time-dependent changes in subjective perceptions of cooling [39]. The Likert scales for thermal sensation, thermal comfort, and thermal acceptance were created based on the recommendations of Schweiker et al. [40], i.e. using a ruler to draw a 100 mm horizonal line the anchors were then spaced evenly along the line. Thermal sensation consisted of a 7-point scale from 1 (cold) to 7 (hot) with 4 as neutral. Thermal comfort used a 5-point scale ranging from 1 (comfortable) to 5 (extremely uncomfortable) and thermal acceptance used a 4-point scale ranging from 1 (clearly acceptable) to 4 (clearly unacceptable). Perceptual sampling will occur at pre-determine time points throughout the entire pressure protocol (Fig 2). This evaluation will establish ageing- and SCI-induced changes in peripheral neurosensory function, as well as the relationship between the physiological and perceptual effects of cooling during mechanical loading.

### Statistical analyses

Data will be assessed for normality of distribution (Kolmogorov—Smirnov test), and then analysed for the independent and interactive effects of pressure stimuli’s temperature (i.e. 3 levels: 38, 24, 16 °C), and of participant group (i.e. 3 levels: young, older, SCI) on within- and between-subject mean differences in peak hyperaemia (N = 35 with 95% Confidence Intervals). This will be conducted using a 2-way mixed model ANOVA or Friedman, depending on the data distribution (parametric or non-parametric, respectively). Post-hoc analyses will be performed between pressure stimuli’s temperatures and participant groups based on the presence of main effects and using Tukey’s test. The use of regression models will evaluate the relationship between absolute cooling temperatures and peak hyperaemia during pressure stimuli. This approach is designed to identify “physiological thresholds” for the protective effects of cooling under mechanical loading. Mean thresholds will subsequently be determined and their inter-individual variability across the cohort. For the perceptual data, thermal discomfort will identify “Uncomfortable” on the Likert Scale as the threshold onset of discomfort. Regression analyses will be used to interrogate the relationship between pressure stimuli’s cooling temperature and local discomfort, for each participant. This approach will also enable the assessment of “perceptual cooling thresholds” for cold-induced discomfort at two absolute cold temperatures (i.e. 24 and 16 °C), and their individual variability. Physiological and perceptual thresholds will be combined to produce common cooling parameters that will accommodate different cooling characteristics. Regarding the secondary experimental outcomes, these will be within- and between-subject changes in biomarkers’ expression, skin structural and biophysical properties (i.e. imaging parameters and skin friction), and subjective thermal perceptions, as a function of pressure stimuli temperature (i.e. 3 levels: 38, 24, 16°C), time (i.e. varying levels depending on variables’ sampling rate), and of participant group (i.e. 3 levels: young, older, SCI). Data will be first assessed for normality of distribution (Kolmogorov—Smirnov test), and then analysed for the independent and interactive effects of pressure and stimuli temperature, time, and participant group by means of a 3- way mixed model ANOVA (or Friedman). Post-hoc analyses will be performed between pressure stimuli temperatures, time, and participant groups based the presence of main effects and using Tukey’s test. Group-related co-variables associated with sex, skin tone, and clinical status (applicable to SCI participants only, e.g. rehabilitation status) will be considered in all analyses to interpret the proportion of variance unexplained by the main effects (i.e. temperature, time, and group) and their interactions.

### Data management

Data management will be in line with the University of Southampton’ policy on data quality, which forms part of the University’s Information Governance Framework and demonstrates compliance with its obligations under the Data Protection Legislation. Therefore, the study will comply with the requirements of the Data Protection Act 2018 and the University of Southampton’s Ethics Committee (ERGO) policies. This project involves human participants and will be conducted in line with the University’s Policy on the Ethical Conduct of Research and Studies involving Human Participants, and the Medical Research Council’s policies on ethics and data sharing. Data will be fully anonymised at the earliest opportunity and before being made available open access in the University’s data repository. All data that supports publications will be deposited and will be citable using a persistent identifier (DOI). Original hardcopies of study documents (e.g. consent forms) will be stored securely for ten years from completion of the project within a locked office at the University or scanned, encrypted and securely stored on the University’s IT system.

### Ethical considerations and declarations

The project will involve testing healthy individuals aged 18 to 70 years, and those with a SCI, and will be conducted in line with Southampton University Code of Practice for Research and will comply with the *Declaration of Helsinki*. Participants will provide written informed consent, and relevant personal information (e.g. skin’s perceptual sensitivity to cooling). All experiments will pose low risks to participants, and not greater than what they face in their daily living (e.g. undergoing a GP examination of skin sensitivity); yet a set of mitigation measures to manage these risks have been developed. For example, there is a risk of discomfort from skin cooling. This will be akin to having a cold pack applied on the skin following an injury. Mitigation measures such as on-going skin temperature monitoring, checks of subjective wellbeing, and active skin re-warming, will be in place. The skin will also be checked for blanching erythema. All research methods for evaluating physiological thermoregulatory and vasomotor responses (e.g. recording of skin temperature via thermocouple microsensors, skin blood flow via Laser Doppler Flowmetry) will be non-invasive (e.g. sensors applied to participants’ skin with hypoallergenic medical tape), and they pose low risk.

Ethical approval for the stated measurements and procedures has been granted by the University of Southampton’s Ethics Committee (ERGO 88984).

### Status and timeline of the study

At the time of publication pilot testing and technical development of the protocol have been completed. Formal recruitment commenced on 16^th^ January 2024. The project is being supported by a Medical Council Research grant (MR/X019144/1) and has a lifespan of 42 months from March 2023.

## Discussion

Skin damage, leading to PUs, can affect any individual who experience prolonged periods of immobility, ranging from newborn babies to older adults. In addition, with the recent Covid-19 pandemic, there has been an enormous increase in the number of hospitalized patients with novel respiratory diseases and the associated healthcare management team, who have developed skin damage from prolonged use of personal protective medical devices [41]. Thus, the proposed research which addresses the physiological tolerance of human skin to prolonged mechanical loading is both important in improving scientific knowledge and timely to societal demands.

### Strengths of the planned study

A purposefully sampled group of human volunteers has the unique advantage of targeting patient-relevant, physiological, and perceptual mechanisms, which would otherwise be inaccessible via in vitro skin constructs or in vivo animal models. Animal studies involving mice and porcine models of mechanically induced PU formation have been developed to investigate how the temperature of loaded skin modulates skin damage [9, 35]. Similarly, in vitro skin models have been used to investigate drug delivery for wound healing [42]. Yet, the translational value of these animal and in vitro models to human participants is limited. This is because differences exist in skin morpho-physiology, immunology, genetics, and thermoregulatory control amongst these models [43]. Importantly, these previous approaches do not allow for the evaluation of the perceptual effects of thermal interventions on loaded skin, critical when considering the adoption in different care settings. Localised skin cooling induces cold discomfort [44], which greatly limits acceptability and adherence to therapeutic interventions designed to maintain skin health, particularly for vulnerable individuals such as the elderly [18]. Hence, a human-centric approach can help identifying optimal levels of cooling that can provide a physiological effect within the acceptable range of perceived comfort. This is critical to develop “user-centred” therapeutic approaches that are both effective and comfortable.

Some animal data indicates that in association with reducing local metabolic demands, reducing skin temperature during applied mechanical loading could preserve metabolic and myogenic components of skin blood flow [11]. This would protect the skin against pressure induced ischemia and reduce the potential of tissue necrosis. Local cooling could also reduce the magnitude of reactive hyperaemia following a period of pressure-induced ischaemia [6]. Reactive hyperaemia can increase the risk of ischemia reperfusion injury by triggering the release of oxygen-derived free radicals with cytotoxic effects, and this can play an equivalent role in the pathophysiology of PUs as sustained pressure [7]. This has been demonstrated with cooling of cardiac tissues to protect from ischemia-reperfusion injury in animal models [45].

This project will generate novel insights on temperature-modulated skin tolerance in vivo, which will be relevant to skin physiologists, bioengineers, and clinicians such as dermatologists and intensive care nurses, to better understand the physiological processes and the potential benefits of cooling strategies to minimise the individual PU risk in a clinical setting.

Establishing the physiological and perceptual relationships between cooling and skin tolerance to pressure will help inform the design of public health interventions to protect vulnerable groups at risk of PUs such as the elderly. The research will improve quality of life in individuals who are at risk of PUs. Improved therapeutic interventions will reduce discomfort and lower the incidence of injuries, thus reducing the financial burden on healthcare providers (cost for NHS wound care ~£8Bn/yr). In addition, effective technologies which provide cooling can be maintained in-situ for prolonged periods, decreasing the demand for repeat interventions. Applications of the physiological and perceptual thresholds will inform the design of user-centred medical devices and wearables, including support surfaces and garments delivering cooling to the skin at a level and rate that is both beneficial and comfortable. This knowledge will be relevant to material and textile engineers engaged in the design of healthcare and medical device products.

### Limitations

The study has been robustly designed but is not without limitation, which relates to the measurement of skin blood flow perfusion and reactive hyperaemia. The measurement of blood perfusion using LDF provides high temporal resolution, however; limited spatial resolution is offered [46], and the optical probe must be placed directly over the area of interest to detect blood flow changes. Thus, the surface area of the optical probe in contact with the skin is limited by the probe size (which is smaller than the thermally stimulated area. Using a single probe could also present limitations due to potential high variability due to a lack of homogeneity in skin morphology and inter-variability of participant anatomy, although single site LDF measurements in the forearm has been shown to be reliable [47]. Future studies should therefore consider methodological advancement to facilitate LDF assessments at multiple sites under concurrent thermal and mechanical stimulation.

## Conclusions

We have designed a clinically relevant set of experiments in healthy young participants and in groups at-risk of PUs, to determine how different levels of cooling alter the skin’ microvascular, inflammatory, structural, and perceptual responses to a) sustained pressure induced ischemia; b) post-occlusive reactive hyperaemia. The outcomes of this project will help identifying the metabolic, immunological, biophysical, and perceptual pathways underlying the potential beneficial effects of cooling on skin tolerance to loading in distinct cohorts to fundamentally change our understanding of normal and pathological skin function. This knowledge will be translated to support innovation of assistive thermal technologies that maintain skin health across the life course.

## References

[1] Kottner J, Cuddigan J, Carville K, Balzer K, Berlowitz D, Law S, et al. Prevention and treatment of pressure ulcers/injuries: The protocol for the second update of the international Clinical Practice Guideline 2019. J Tissue Viability. 2019;28(2):51–8. doi: 10.1016/j.jtv.2019.01.001 30658878

[2] Guest JF, Fuller GW, Vowden P. Cohort study evaluating the burden of wounds to the UK’s National Health Service in 2017/2018: update from 2012/2013. BMJ Open. 2020;10(12):e045253. doi: 10.1136/bmjopen-2020-045253 33371051 PMC7757484

[3] Coleman S, Nixon J, Keen J, Wilson L, McGinnis E, Dealey C, et al. A new pressure ulcer conceptual framework. J Adv Nurs. 2014;70(10):2222–34. doi: 10.1111/jan.12405 24684197 PMC4263098

[4] Kottner J, Black J, Call E, Gefen A, Santamaria N. Microclimate: A critical review in the context of pressure ulcer prevention. Clin Biomech (Bristol, Avon). 2018;59:62–70. doi: 10.1016/j.clinbiomech.2018.09.010 30199821

[5] Worsley PR, Crielaard H, Oomens CWJ, Bader DL. An evaluation of dermal microcirculatory occlusion under repeated mechanical loads: Implication of lymphatic impairment in pressure ulcers. Microcirculation. 2020;27(7):e12645. doi: 10.1111/micc.12645 32603524

[6] Tzen YT, Brienza DM, Karg PE, Loughlin PJ. Effectiveness of local cooling for enhancing tissue ischemia tolerance in people with spinal cord injury. J Spinal Cord Med. 2013;36(4):357–64. doi: 10.1179/2045772312Y.0000000085 23820151 PMC3758532

[7] Tsuji S, Ichioka S, Sekiya N, Nakatsuka T. Analysis of ischemia-reperfusion injury in a microcirculatory model of pressure ulcers. Wound Repair Regen. 2005;13(2):209–15. doi: 10.1111/j.1067-1927.2005.130213.x 15828947

[8] Clark M. Microclimate: Rediscovering an Old Concept in the Aetiology of Pressure Ulcers. In: Romanelli M, Clark M, Gefen A, Ciprandi G, editors. Science and Practice of Pressure Ulcer Management. London: Springer London; 2018. p. 103–10.

[9] Kokate JY, Leland KJ, Held AM, Hansen GL, Kveen GL, Johnson BA, et al. Temperature-modulated pressure ulcers: a porcine model. Arch Phys Med Rehabil. 1995;76(7):666–73. doi: 10.1016/s0003-9993(95)80637-7 7605187

[10] Iaizzo PA, Kveen GL, Kokate JY, Leland KJ, Hansen GL, Sparrow EM. Prevention of pressure ulcers by focal cooling: Histological assessment in a porcine model. Wounds. 1995;7:161–9.

[11] Jan YK, Lee B, Liao F, Foreman RD. Local cooling reduces skin ischemia under surface pressure in rats: an assessment by wavelet analysis of laser Doppler blood flow oscillations. Physiol Meas. 2012;33(10):1733–45. doi: 10.1088/0967-3334/33/10/1733 23010955 PMC3534840

[12] Lee Bernard and Benyajati Siribhinya and Woods Jeffrey A and Jan Yih-Kuen. Effect of local cooling on pro-inflammatory cytokines and blood flow of the skin under surface pressure in rats: Feasibility study. Journal of Tissue Viability. 2014;23(2):69–77. 24513091 10.1016/j.jtv.2014.01.002

[13] Lachenbruch C. Skin cooling surfaces: estimating the importance of limiting skin temperature. Ostomy Wound Manage. 2005;51(2):70–9. 15699555

[14] Bergstrand S, Lindberg LG, Ek A-C, Lindén M, Lindgren M. Blood flow measurements at different depths using photoplethysmography and laser Doppler techniques. Skin Research and Technology. 2009;15(2):139–47. doi: 10.1111/j.1600-0846.2008.00337.x 19622122

[15] Tzen YT, Brienza DM, Karg P, Loughlin P. Effects of local cooling on sacral skin perfusion response to pressure: implications for pressure ulcer prevention. J Tissue Viability. 2010;19(3):86–97. doi: 10.1016/j.jtv.2009.12.003 20149965

[16] Lee B, Benyajati S, Woods JA, Jan YK. Effect of local cooling on pro-inflammatory cytokines and blood flow of the skin under surface pressure in rats: feasibility study. J Tissue Viability. 2014;23(2):69–77. doi: 10.1016/j.jtv.2014.01.002 24513091

[17] Wang W, Wang H, Zhao Z, Huang X, Xiong H, Mei Z. Thymol activates TRPM8-mediated Ca(2+) influx for its antipruritic effects and alleviates inflammatory response in Imiquimod-induced mice. Toxicol Appl Pharmacol. 2020;407:115247. doi: 10.1016/j.taap.2020.115247 32971067

[18] Ledger L, Worsley P, Hope J, Schoonhoven L. Patient involvement in pressure ulcer prevention and adherence to prevention strategies: An integrative review. Int J Nurs Stud. 2020;101:103449. doi: 10.1016/j.ijnurstu.2019.103449 31706155

[19] Filingeri D, Redortier B, Hodder S, Havenith G. Thermal and tactile interactions in the perception of local skin wetness at rest and during exercise in thermo-neutral and warm environments. Neuroscience. 2014;258:121–30. doi: 10.1016/j.neuroscience.2013.11.019 24269934

[20] Worsley PR, Bader DL. A modified evaluation of spacer fabric and airflow technologies for controlling the microclimate at the loaded support interface. Textile Research Journal. 2019;89(11):2154–62.

[21] van Leen M, Halfens R, Schols J. Preventive Effect of a Microclimate-Regulating System on Pressure Ulcer Development: A Prospective, Randomized Controlled Trial in Dutch Nursing Homes. Adv Skin Wound Care. 2018;31(1):1–5. doi: 10.1097/01.ASW.0000527288.35840.0a 29240594

[22] Denzinger M, Rothenberger J, Held M, Joss L, Ehnert S, Kolbenschlag J, et al. A quantitative study of transepidermal water loss (TEWL) on conventional and microclimate management capable mattresses and hospital beds. J Tissue Viability. 2019;28(4):194–9. doi: 10.1016/j.jtv.2019.06.002 31272882

[23] Holbrook KA, Odland GF. Regional differences in the thickness (cell layers) of the human stratum corneum: an ultrastructural analysis. J Invest Dermatol. 1974;62(4):415–22. doi: 10.1111/1523-1747.ep12701670 4820685

[24] Johnson JM, Minson CT, Kellogg DL. Cutaneous vasodilator and vasoconstrictor mechanisms in temperature regulation. Compr Physiol. 2014;4(1):33–89. doi: 10.1002/cphy.c130015 24692134

[25] Forsyth P, Miller J, Pumpa K, Thompson KG, Jay O. Independent Influence of Spinal Cord Injury Level on Thermoregulation during Exercise. Med Sci Sports Exerc. 2019;51(8):1710–9. doi: 10.1249/MSS.0000000000001978 30865188

[26] Zeilig G, Enosh S, Rubin-Asher D, Lehr B, Defrin R. The nature and course of sensory changes following spinal cord injury: predictive properties and implications on the mechanism of central pain. Brain. 2012;135(Pt 2):418–30. doi: 10.1093/brain/awr270 22094538

[27] Lorenzo S, Minson CT. Human cutaneous reactive hyperaemia: role of BKCa channels and sensory nerves. J Physiol. 2007;585(Pt 1):295–303. doi: 10.1113/jphysiol.2007.143867 17901123 PMC2375471

[28] De Jong RH, Hershey WN, Wagman IH. Nerve conduction velocity during hypothermia in man. Anesthesiology. 1966;27(6):805–10. doi: 10.1097/00000542-196611000-00013 5924554

[29] Fitzpatrick Thomas. Soleil et peau [Sun and skin]. J Méd Esthétique. 1975;2:33–4.

[30] Fawcett JW, Curt A, Steeves JD, Coleman WP, Tuszynski MH, Lammertse D, et al. Guidelines for the conduct of clinical trials for spinal cord injury as developed by the ICCP panel: spontaneous recovery after spinal cord injury and statistical power needed for therapeutic clinical trials. Spinal Cord. 2007;45(3):190–205. doi: 10.1038/sj.sc.3102007 17179973

[31] Urbaniak GC, Plous S. Resaerch Randomizer (Version 4.0) [Computer software] 2013 [http://www.randomizer.org.

[32] Jayabal H, Bader DL, Worsley P. Development of an Efficient Extraction Methodology to Analyse Potential Inflammatory Biomarkers from Sebum. Skin Pharmacol Physiol. 2023;36(1):38–50. doi: 10.1159/000528653 36572004

[33] Humeau A, Steenbergen W, Nilsson H, Strömberg T. Laser Doppler perfusion monitoring and imaging: novel approaches. Med Biol Eng Comput. 2007;45(5):421–35. doi: 10.1007/s11517-007-0170-5 17340155

[34] Wright CI, Kroner CI, Draijer R. Non-invasive methods and stimuli for evaluating the skin’s microcirculation. J Pharmacol Toxicol Methods. 2006;54(1):1–25. doi: 10.1016/j.vascn.2005.09.004 16256378

[35] Lachenbruch C, Tzen YT, Brienza DM, Karg PE, Lachenbruch PA. The relative contributions of interface pressure, shear stress, and temperature on tissue ischemia: a cross-sectional pilot study. Ostomy Wound Manage. 2013;59(3):25–34. 23475449

[36] Hoogendoorn I, Reenalda J, Koopman B, Rietman JS. The effect of pressure and shear on tissue viability of human skin in relation to the development of pressure ulcers: a systematic review. J Tissue Viability. 2017;26(3):157–71. doi: 10.1016/j.jtv.2017.04.003 28457615

[37] Soetens JFJ, Worsley PR, Bader DL, Oomens CWJ. Investigating the influence of intermittent and continuous mechanical loading on skin through non-invasive sampling of IL-1α. J Tissue Viability. 2019;28(1):1–6. 30638732 10.1016/j.jtv.2018.12.003

[38] Adan F, Nelemans PJ, Essers BAB, Brinkhuizen T, Dodemont SRP, Kessels J, et al. Optical coherence tomography versus punch biopsy for diagnosis of basal cell carcinoma: a multicentre, randomised, non-inferiority trial. Lancet Oncol. 2022;23(8):1087–96. doi: 10.1016/S1470-2045(22)00347-3 35835136

[39] Typolt O, Filingeri D. Evidence for the involvement of peripheral cold-sensitive TRPM8 channels in human cutaneous hygrosensation. Am J Physiol Regul Integr Comp Physiol. 2020;318(3):R579–r89. doi: 10.1152/ajpregu.00332.2019 31967850

[40] Schweiker M, André M, Al-Atrash F, Al-Khatri H, Alprianti RR, Alsaad H, et al. Evaluating assumptions of scales for subjective assessment of thermal environments—Do laypersons perceive them the way, we researchers believe? Energy and Buildings. 2020;211:109761.

[41] Évora SA, Abiakam N, Jayabal H, Worsley PR, Zhang Z, S AJ, et al. Characterisation of superficial corneocytes in skin areas of the face exposed to prolonged usage of respirators by healthcare professionals during COVID-19 pandemic. J Tissue Viability. 2023;32(2):305–13. doi: 10.1016/j.jtv.2023.02.007 36813598 PMC9918437

[42] Ud-Din S, Bayat A. Non-animal models of wound healing in cutaneous repair: In silico, in vitro, ex vivo, and in vivo models of wounds and scars in human skin. Wound Repair Regen. 2017;25(2):164–76. doi: 10.1111/wrr.12513 28120405

[43] Zomer HD, Trentin AG. Skin wound healing in humans and mice: Challenges in translational research. J Dermatol Sci. 2018;90(1):3–12. doi: 10.1016/j.jdermsci.2017.12.009 29289417

[44] Cotter JD, Taylor NA. The distribution of cutaneous sudomotor and alliesthesial thermosensitivity in mildly heat-stressed humans: an open-loop approach. The Journal of physiology. 2005;565:335–45. doi: 10.1113/jphysiol.2004.081562 15760945 PMC1464483

[45] Olivecrona GK, Götberg M, Harnek J, Van der Pals J, Erlinge D. Mild hypothermia reduces cardiac post-ischemic reactive hyperemia. BMC Cardiovasc Disord. 2007;7:5. doi: 10.1186/1471-2261-7-5 17324251 PMC1808476

[46] Strömberg T, Sjöberg F, Bergstrand S. Temporal and spatiotemporal variability in comprehensive forearm skin microcirculation assessment during occlusion protocols. Microvasc Res. 2017;113:50–5. doi: 10.1016/j.mvr.2017.04.005 28455225

[47] Roustit M, Blaise S, Millet C, Cracowski JL. Reproducibility and methodological issues of skin post-occlusive and thermal hyperemia assessed by single-point laser Doppler flowmetry. Microvasc Res. 2010;79(2):102–8. doi: 10.1016/j.mvr.2010.01.001 20064535

